# By Regulating the NLRP3 Inflammasome Can Reduce the Release of Inflammatory Factors in the Co-Culture Model of Tuberculosis H37Ra Strain and Rat Microglia

**DOI:** 10.3389/fcimb.2021.637769

**Published:** 2021-04-13

**Authors:** Zhen Xie, Hao Hui, Qian Yao, Yan Duan, Wu Li, Ye Cheng, Meng Zhang, Ye Tian, Gang Zhao

**Affiliations:** ^1^ The College of Life Sciences and Medicine, Northwest University, Xi’an, China; ^2^ Department of Neurology, Department of Medical Research Center, Xi’an Key Laboratory of Cardiovascular and Cerebrovascular Diseases, Xi’an NO.3 Hospital, The Affiliated Hospital of Northwest University, Xi’an, China; ^3^ Department of Spine Surgery, Honghui Hospital, The Affiliated Hospital of Xi’an Jiaotong University, Xi’an, China

**Keywords:** CNS tuberculosis infection, microglia, NLRP3 inflammasome, caspase-1, NF-κB, P2X7 receptor

## Abstract

**Objective:**

Tuberculosis infection of the Central Nervous System can cause severe inflammation in microglia, and NLRP3 inflammasome is also an important source of inflammation in microglia. Therefore, in this study, we used a co-culture model of rat microglia and tuberculosis H37Ra strain to explore the influence of tuberculosis infection on the NLRP3 inflammasome in microglia and its regulation mechanism.

**Methods:**

We cultured primary microglia from SD rats and co-cultured with tuberculosis H37Ra strain for 4 hours to establish a co-culture model. At the same time, MCC950, Z-YVAD-FMK, BAY-11-7082, Dexamethasone, RU486, BzATP, BBG and extracellular high potassium environment were used to intervene the co-cultivation process. Subsequently, western blot, real-time PCR, ELISA and other methods were used to detect the changes of NLRP3 inflammasome-related molecules in microglia.

**Results:**

After co-cultivation, the NLRP3 inflammasomes in microglia were activated and released a large amount of IL-18 and IL-1β. By regulating NLRP3 inflammasome complex, caspase-1, NF-κB and P2X7R during the co-culture process, it could effectively reduce the release of IL-18 and IL-1β, and the mortality of microglia.

**Conclusion:**

Our results indicate that the NLRP3 inflammasome pathway is an important part of the inflammatory response of microglia caused by tuberculosis infection. By intervening the NLRP3 inflammasome pathway, it can significantly reduce the inflammatory response and mortality of microglia during the tuberculosis H37Ra strain infection. This research can help us further understand the inflammatory response mechanism of the central nervous system during tuberculosis infection and improve its treatment.

## Introduction

Central Nervous System (CNS) tuberculosis infection is a very serious disease caused by Mycobacterium tuberculosis (*Mtb*), with a fatality rate of 20% and a disability rate of 50% (Hernandez Pando, 2011). In the CNS, microglia are one of the immune cells against *Mtb (* Rock et al., 2004). In this process, microglia produce a large number of inflammatory factors (Rock et al., 2004). And excessive inflammation usually leads to a poor prognosis (Hernandez Pando, 2011). Therefore, studying the mechanism of excessive inflammation caused by microglia and its intervention factors in the process of Mtb infection in the central nervous system is of great significance for improving the treatment and prognosis of the disease.

Microglia are the most important myeloid cells in healthy brain parenchyma, and they play many important functions such as homeostatic monitoring and immune defense (Wolf et al., 2017). Microglia originate from primitive macrophages, then leave the yolk sac and colonize in the neuroepithelium, and become resident macrophages in CNS (Prinz et al., 2017). Therefore, the properties and functions of microglia and macrophages are very similar (Prinz et al., 2017). Previous studies indicate that the baseline level of NLRP3 inflammasomes in macrophages is very low, but after *Mtb* infection, the NLRP3 inflammasomes in macrophages are activated, meanwhile, the secretion of various inflammatory cytokines such as IL-18 and IL-1β increase significantly (Song and Li, 2018). In addition, when a variety of pathogens infect the CNS, the activation of NLRP3 inflammasomes also occurs in microglia (Dong et al., 2020; Ma et al., 2020; Wang S et al., 2020). Therefore, we speculated that when *Mtb* infects the CNS, the NLRP3 inflammasome in microglia might also be activated, and cause the microglia to secrete a variety of inflammatory factors.

NLRP3 inflammasome is a typical inflammasome that can respond to various danger signals inside and outside the cell, it represents a special cell death process called pyroptosis, which can occur in a variety of infections and inflammatory diseases (Wang Z et al., 2020). Similarly, the involvement of NLRP3 inflammasome can also be observed in various CNS diseases such as infection, ischemia, and degeneration (Marim et al., 2017; Heneka et al., 2018; Katuri et al., 2019; Xue et al., 2019; Cui et al., 2020; Ji et al., 2020; Ma et al., 2020; Zeng et al., 2020). The activation of NLRP3 inflammasome is a two-stage process. The first stage (signal 1) is the sensing and generation stage, which starts with the recognition of PAMP and DAMP by TLR. At this stage, TLR recognizes various stimulus signals and factors and activates the NF-κB signaling pathway, leading to an increase in the transcription and translation of a variety of precursor proteins (Shao et al., 2019). The second stage (signal 2) is the assembly and effector stage, which starts with the assembly of the NLRP3 inflammasome. NLRP3, ASC and pro-caspase-1 assemble into a mature inflammasome complex, and convert pro-caspase-1 into caspase-1, then caspase-1 convert pro-IL-1β and pro-IL-18 into their mature forms (Swanson et al., 2019). IL-1β and IL-18 can cause subsequent inflammation. The regulation of cell pyroptosis mainly focuses on signal 1 and signal 2 pathways. For example, the NF-κB in signal 1 is an important nuclear transcription factor. Interfering with the phosphorylation level of NF-κB upstream molecules can affect the transfer of NF-κB to the nucleus, thereby affecting the transcription of many molecules in the nucleus (Irrera et al., 2017). The P2X7 receptor (P2X7R) in signal 2 is a kind of non-specific positive transcription in the nucleus, it is widely distributed in microglia and other cells (Su et al., 2018), belonging to the P2X subfamily of purinergic P2 receptor ligand-gated ion channels. It can be stimulated by a large amount of extracellular ATP released by dying cells, or activated by outflow of large amounts of potassium ions, thereby triggering various signal cascades and promoting the assembly of NLRP3 inflammasome complex and the expression of related molecules (Karmakar et al., 2016). Recently, researchers have also found a protein called Gasdermin D, which is located downstream of caspase-1 and is called the executor of pyroptosis. It is necessary for the secretion of IL-1β (He et al., 2015).

Therefore, in this experiment, we used a co-culture model of rat primary microglia and *Mtb* H37Ra strain to simulate CNS *Mtb* infection. In this model, we studied whether there is activation of NLRP3 inflammasome pathways in microglia, and initially explored its possible intervention factors and mechanisms. This research can help us understand the mechanism of inflammatory response and its intervention factors after *Mtb* infection in the CNS, and it is of great significance to improve the treatment and prognosis of the disease.

## Reagents

Pubs of SD rats were purchased from Northwestern University Animal Experiment Center. Dimethylsulfoxide (DMSO), MTT, Cytotoxicity Detection Kit-LDH, poly-L-lysine, and BCA protein quantification kit were from Beyotime, CN. P2X7R antibody, NLRP3 antibody, pro-IL-18 antibody, GSDMD antibody, CD11b antibody were from abcam, UK. Pro-IL-1β antibody, Caspase-1 (P10) antibody, pro-caspase-1(P45) antibody, and β-actin antibody, β-tubulin antibody were from Santa Cruz Biotechnology, CA. Phospho-NF-κB p65 antibody and Phospho-IκB-α were from Cell Signal Technology, USA. Serum IL-1β and IL-18 ELISA kit, BAY11-7082, RU486, Z-YVAD-FMK, TRIzol Reagent were from Sigma, USA. Revert Aid First Strand cDNA Synthesis Kit was from Thermo, USA. SYBR was from Takara, JP. GAPDH was from Sangon, CN. DEX was from Henan Runhong Pharmaceutical Co., Ltd., CN and Nicotine was from Shanghai Shuoguang Electronics Co., Ltd. *Mtb* H37Ra stain was from Shanghai Kanglang Biotechnology Co., Ltd., CN. Löwenstein-Jensen slope was from Solarbio, CN. Middlebrool 7H9 broth base was from BinSuiBio, CN. Nuclear and Cytoplasmic Extraction Kit was from CWBIO, CN (See the **Supplementary Files** for details).

## Materials and Methods

### Primary Microglia Culture

Primary microglia were obtained from SD rat pups within 4 hours of postnatal. After hypothermic anesthesia, the pups were decapitated and their brains were excised. Peeled off the meninges of the brain tissue, and then immersed it in 0.25% trypsin-EDTA, then mechanically separated the brain tissue with a 25-gauge needle, and homogenized it with a manual homogenizer. The cell suspension was seeded into poly-L-lysine-coated T150 tissue culture flasks and maintained in DMEM/F12 with 10% FBS and 1% penicillin-streptomycin for 10–14 days to grow a confluent mixed astrocyte/microglia population. On the 16th day, the cells were collected, positively selected by the antibody CD11b, and separated using a magnetic column combined with a magnetic ball compounded to the CD11b surface marker. Finally, the cells were transplanted into a six-well plate, adjusted the number of cells per well to about 1*10^5^, and follow-up experiments were carried out 48 hours later. Microglial cell specific antibody CD11b was used for immunohistochemical staining. The microglial cell cultures used were >95% pure.

### Cultivate *Mtb* H37Ra Strain

*Mtb* H37Ra stain was inoculated on the Löwenstein-Jensen slope for two weeks. The bacteria were then transferred to 7H9-S medium (Middlebrool 7H9 broth base, with 10% ADC-albumin glucose catalase and 0.2% glycerol), and were incubated for half an hour after shaking. Finally, the supernatant was placed in a clean test tube, and adjusted its final density to approximately 1×10^6^ CFU/ml with 7H9-S broth (confirm the inoculum concentration by counting CFU on the agar plate).

### Co-Culture of Microglia and H37Ra Strain

We co-cultured microglia with the tuberculosis H37Ra strain to establish a co-culture model. After the microglia were cultured in a six-well plate for 48 hours, 0.1ml of H37Ra strain at 1×10^6^ CFU/ml was added to each well. In addition, different intervention factors were added to the wells, including MCC950 (10μM), Z-YVAD-FMK (50μM), BAY-11-7082 (10μM), RU486 (10μM), dexamethasone (DEX, 10μM), Potassium ion solution (K+, adjust the concentration to 150 mM), BBG (100nM), BzATP (100μM). After 4 hours of co-culture, we collected the cells and supernatant separately. Then, we washed the collected cells 3 times with PBS, and centrifuged the collected cell culture solution to remove bacteria and cells for subsequent experiments.

### Western Blotting

In short, a modified RIPA buffer was used to lyse and extract cellular proteins. Nuclear and Cytoplasmic Extraction Kit was used to extract nuclear proteins. Then the extracted cell protein was quantified with BCA Protein Assay Kit. An equal amount of protein was loaded into each well of a 10% SDS-acrylamide gel, separated by gel electrophoresis, and then transferred to a nitrocellulose membrane. At room temperature, 5% skim milk blocking buffer was used to block the nitrocellulose membrane for 1 h. Then the membrane was incubated with the primary antibody overnight at 4°C. The next day, after washing three times with PBS, the membrane was incubated with a peroxidase-linked secondary antibody for 1 hour at room temperature. Subsequently, the bands were visualized by chemiluminescence (Millipore). ImageJ (NIH, USA) was used to analyze the intensity of each band. β-actin and β-tubulin were used as internal control.

### ELISA to Detect Released Cytokines

After the co-cultivation, the extracellular medium was removed from each well and centrifuged at 10,000×g for 15 seconds to precipitate the cells, bacteria and other impurities. Then the concentration of IL-1β and IL-18 in the supernatant was determined according to the ELISA kit.

### Real-Time PCR

After co-cultivation, the microglia cells were washed three times, and then used TRIzol Reagent to prepare total RNA according to the manufacturer’s protocol (Invitrogen Life Technologies). As RevertAidTM First Strand cDNA Synthesis Kit, one microgram of total RNA in each sample was used to generate cDNA. Amplify the resulting cDNA with SYBR. The process was detected by CFX96 Touch TM real-time PCR detection system. Finally, the 2-ΔΔCt method was used for relative quantitative analysis of mRNA. The results obtained are normalized to GAPDH. Real-time polymerase chain reaction used the following primers:

p2x7r: Forward primer 5′-CTACTCTTCGGTGGGGGCTT-3′

Reverse primer 5′-CTCTGGATCCGGGTGACTTT-3′

nlrp3: forward primer 5’-GAGCTGGACCTCAGTG ACAATGC-3’

Reverse primer 5’-ACCAATGCGAGATCCTG ACAACAC-3’

caspase-1: Forward primer 5’-AGGAGGGAATATGTGG G-3’

Reverse primer 5’-AACCTTGGGCTTGTCT T-3’

gapdh: Forward primer 5’-GACATGCCGCCTGGA GAAAC-3’

Reverse primer 5’-AGCCCAGGATGCCCTT TAGT-3’

il-1β: forward primer: 5′-CCCTGAACTCAACTGTGAAATAGCA-3′,

Reverse primer: 5′-GACTGGCTGTGACCCTATCTGTGA-3′;

il-18: Forward primer 5’-GACTGGCTGTGACCCTATCTGTGA-3’

Reverse primer 5’-TTGTGTCCTGGCACACGTTTC-3’

### Cytotoxicity Determination (LDH Release)

After co-cultivation, the extracellular medium was removed from each well and centrifuged at 10,000×g for 15 seconds to pellet the cells and bacteria. Then the Cytotoxicity Detection Kit-LDH was used to determine the lactate dehydrogenase enzyme (LDH) in the supernatant.

### MTT Analysis to Assess Cell Viability

After the co-cultivation was completed, discarded the original culture medium in each well and washed with PBS for three times. Then added 100μL of the prepared MTT (0.5 mg/ml) and incubate in a 5% CO2 incubator at 37°C for 4 hours. Discarded the supernatant, added 100 μL DMSO to each well, shaked well for 10 min to dissolve the crystals, and measured the absorbance at 570 nm with a microplate reader.

### Statistical Analysis

All experimental results were obtained after performing at least 3 times the same experiment. All data were presented as mean ± standard deviation (SD) of independent experiments. Choose spss20.0 as the statistical analysis software. Two-tailed one-way analysis of variance (ANOVA) with multiple comparison *post hoc* analysis was used, P value<0.05 was considered statistically significant.

## Result

### The Co-Culture of H37Ra and Microglia Could Activate the NLRP3 Inflammasome in Microglia and Promote the Release of Inflammatory Factors Such as IL-18 and IL-1β

(1) NLRP3 inflammasome pathway in microglia could be activated by *Mtb* H37Ra, and the drugs used in this experiment had no significant effect on microglia.

**Figure 1:**
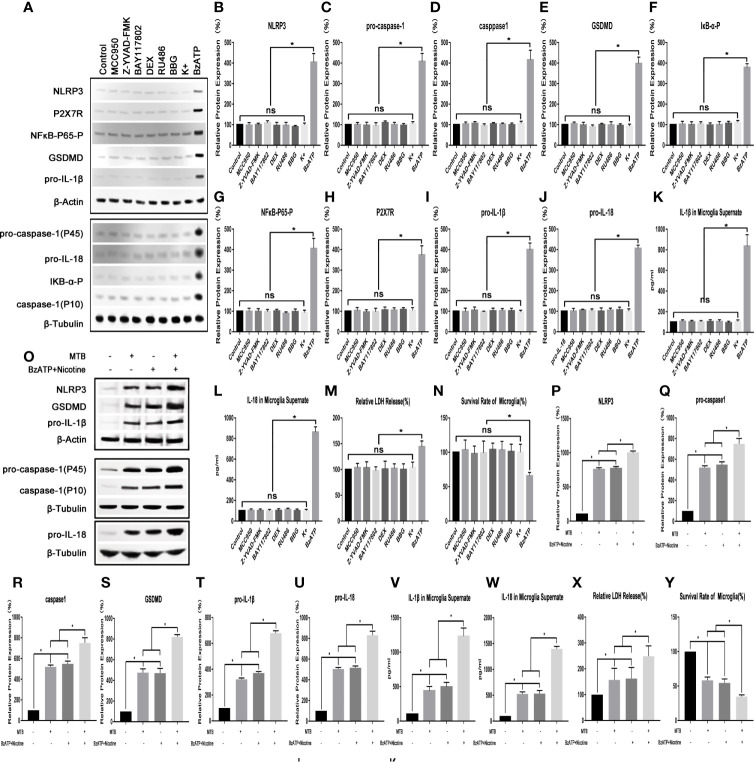
Observe the changes of NLRP3 inflammasome pathway and survival rate of microglia. **(A–N)** The influence of various inhibitors and agonists on microglia. Except for BzATP, the related drugs used in this experiment have no significant effect on the NLRP3 inflammasome pathway and survival rate of microglia under normal conditions. **(O–Y)** The effect of Mtb H37Ra and nicotine+BzATP on the NLRP3 inflammasome pathway in microglia. NLRP3 inflammasome pathway in microglia can be activated by the Mtb H37Ra strain, and its effect is similar to nicotine + BzATP, and the effect is more obvious when the two are used in combination. (*P < 0.05. ns, no significant difference).

As shown in **Figure 1**, we first applied a strong activator of the NLRP3 inflammasome pathway (nicotine (100 mM) + BzATP (100 mM)) as a control to verify whether the NLRP3 inflammasome pathway in microglia could be activated by *Mtb* H37Ra. At the same time, we also observed whether the relevant inhibitors and activators in this experiment could affect the state of microglia. The results showed that the NLRP3 inflammasome pathway in microglia could be activated by the *Mtb* H37Ra strain, and its effect was similar to nicotine +BzATP, and the effect was more obvious when the two were used in combination. After *Mtb* H37Ra strain or/and nicotine+BzAPT were used on microglia, the expression of NLRP3 inflammasome, pro-caspase-1, caspase-1, GSDMD, pro-IL-1β, pro-IL -18, etc. were significantly increased, and the contents of IL-1β and IL-18 in the supernate were obvious increasing, the release rate of LDH was eventually increased, and the survival rate of microglia was decreased. Subsequently, we observed the effects of related inhibitors and agonists that may be used in this experiment on microglia. The results showed that the inhibitor MCC950, Z-YVAD-FMK, BAY117802, DEX, RU486, BBG and KCl had no significant effect on the survival and activation of the NLRP3 inflammatory pathway of microglia. However, the agonist BzATP could activate the NLRP3 inflammasome pathway in microglia to a certain extent, leading to increased secretion of IL-1β and IL-18, and the increased release rate of LDH, and also decreased survival rate of microglia at the same time.

(2) After co-cultivation, the expression of NLRP3 inflammasome, caspase-1, IL-18 and IL-1β in microglia were up-regulated.

**Figure 2:**
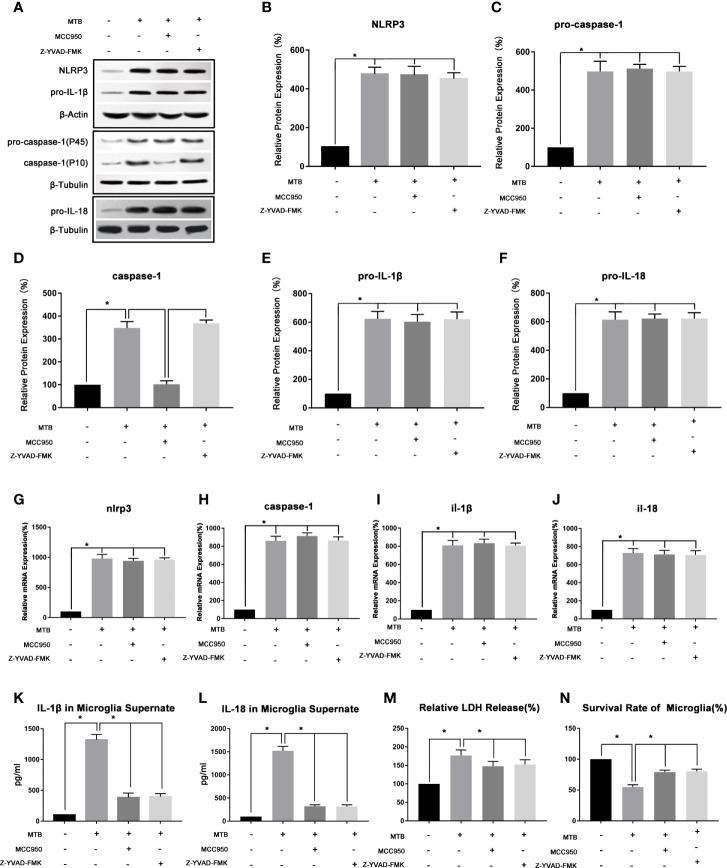
Observe the changes of NLRP3 inflammasome of microglia in the co-culture model. **(A–F)** Use Western Blot to detect the relative protein expression of NLRP3, pro-caspase-1, caspase-1, pro-IL-1β and pro-IL-18 in control group, co-culture group, MCC950 group and Z-YVAD-FMK group. **(G–J)** Use Real-time PCR to detect the relative mRNA expression of nlrp3, caspase-1, il-1β and il-18 in control group, co-culture group, MCC950 group and Z-YVAD-FMK group. **(K, L)** Use ELISA to detect the IL-1β and IL-18 secretion in supernatant of control group, co-culture group, MCC950 group and Z-YVAD-FMK group. **(M)** Use LDH release to measure the cytotoxicity in control group, co-culture group, MCC950 group and Z-YVAD-FMK group. **(N)** Use MTT analysis to detect the cell survival rate in control group, co-culture group, MCC950 group and Z-YVAD-FMK group. *P < 0.05.

As show in **Figure 2**, after co-cultivated microglia and *Mtb* H37Ra strain for 4 hours, we collected the cells and supernatant, and observed the changes of NLRP3, caspase-1, IL-18, and IL-1β in microglia in terms of transcription, protein expression, and inflammatory factor secretion. Experimental results showed that after co-culture, the NLRP3 inflammasome pathway in microglia was activated, and the secretion of LDH, IL-18 and IL-1β in the culture supernatant was also significantly increased. In addition, the survival rate of microglia decreased significantly. Compared with control group, in microglia of co-culture group, the mRNA and protein content of NLRP3 increased significantly (P<0.05). The mRNA content of caspase-1, IL-1β and IL-18, as well as the protein content of pro-caspase-1, pro-IL-18, pro-IL-1β, and activated caspase-1 were increased significantly (P <0.05). And the levels of IL-18 and IL-1β in the supernatant was also increased significantly (P<0.05). Finally, we measured the LDH content and cell survival rate in microglia after co-culture, and found that the LDH content increased significantly (P<0.05), while the cell survival rate decreased significantly (P<0.05).

(3) Intervening the co-culture process with MCC950 could inhibit the function of NLRP3 inflammasome, reduce the release of IL-18 and IL-1β, and increase the survival of microglia.

As show in **Figure 2**, we used the NLRP3 inhibitor MCC950 to interfere with the co-cultivation process. After 4 hours of co-cultivation, the microglia and supernatant were collected, and the NLRP3 inflammasome-related moleculars in the microglia were detected, as well as the secretion of IL-18 and IL-1β. Compared with the co-culture group, the moleculars in microglia in MCC950 intervention group changed as follow, the mRNA and protein content of NLRP3 did not change significantly (P>0.05), as well as the mRNA content of caspase-1 and the protein content of pro-caspase-1 (P>0.05). However, the protein content of activated caspase-1 was significantly reduced (P <0.05). Then we observed the mRNA content of IL-1β and IL-18, as well as the protein content of pro-IL-18 and pro-IL-1β, and found that there was also no significant change (P>0.05), but the content of IL-18 and IL-1β in the supernatant was significantly reduced (P<0.05). Finally, we measured the LDH content of microglia and the cell survival rate. Compared with the co-culture group, the LDH content of microglia in the MCC950 intervention group decreased and the cell survival rate was significantly improved (P<0.05).

(4) Z-YVAD-FMK could inhibit the function of caspase-1, thereby reducing the production of IL-18 and IL-1β in microglia.

As shown in **Figure 2**, we then added Z-YVAD-FMK, the inhibitor of caspase-1, during the co-cultivation process. After 4 hours of co-cultivation, the cells and supernatant were collected separately. Subsequently, the changes of NLRP3, caspase-1, IL-18, and IL-1β in microglia and supernatant were detected. The results showed that in Z-YVAD-FMK inhibitor group, compared with the co-culture group, the mRNA content of nlrp3, caspase-1, il-18, il-1β, and the protein content of NLRP3, pro-caspase-1, caspase-1, pro-IL-18 and pro-IL-1β were not significantly changed (P>0.05). However, IL-1β and IL-18 in the supernatant were significantly reduced (P<0.05), and the content of LDH in microglia decreased and the cell survival rate were increased (P<0.05).

### During the Co-Cultivation, the Activation of NLRP3 Inflammasome in Microglia Involved NF-κB Pathway

(1) After co-culture, NLRP3 inflammasomes in microglia could be activated through the NF-κB pathway. By using BAY-11-7082 to inhibit IκB-α, the activation of NLRP3 could be inhibited.

**Figure 3:**
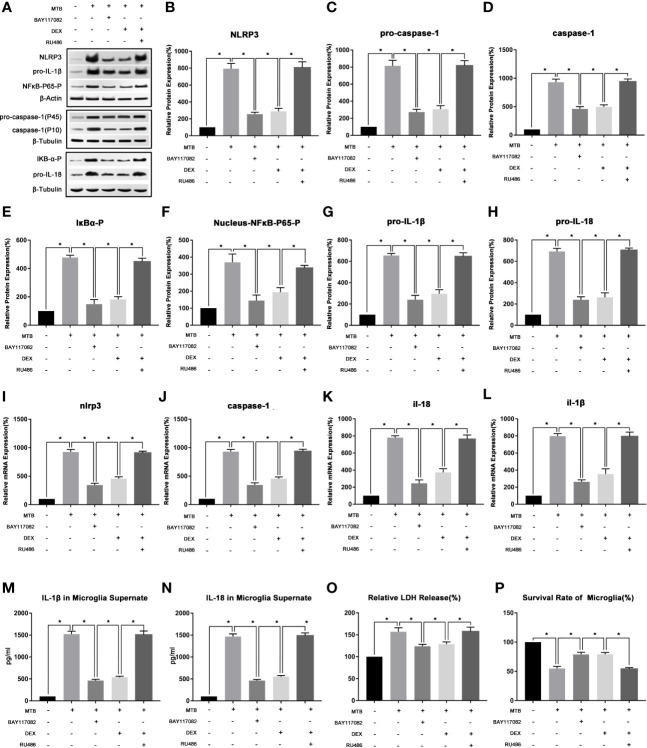
The changes of NF-κB pathway and its intervention factors during the activation of NLRP3 inflammasome in microglia. **(A–H)** Use Western Blot to detect the relative protein expression of NLRP3, pro-caspase-1, caspase-1, pro-IL-1β and pro-IL-18, phosphorylated IκB-α and phosphorylated NF-κB-P65 in control group, co-culture group, BAY-11-7082 group, DEX group and RU486+DEX group. **(I–L)** Use Real-time PCR to detect the relative mRNA expression of nlrp3, caspase-1, il-1β and il-18 in control group, co-culture group, BAY-11-7082 group, DEX group and RU486+DEX group. **(M, N)** Use ELISA to detect the IL-1β and IL-18 secretion in supernatant of control group, co-culture group, BAY-11-7082 group, DEX group and RU486+DEX group. **(O)** Use LDH release to measure the cytotoxicity in control group, co-culture group, BAY-11-7082 group, DEX group and RU486+DEX group. **(P)** Use MTT analysis to detect the cell survival rate in control group, co-culture group, BAY-11-7082 group, DEX group and RU486+DEX group. *P < 0.05.

As show in **Figure 3**, we first examined the expression levels of phosphorylated IκB-α in microglia and phosphorylated NF-κB-P65 in nucleus in microglia after co-culture. The results showed that compared with control group, the level of phosphorylated IκB-α in microglia and phosphorylated NF-κB-P65 in its nucleus of the co-culture group were significantly increased (P<0.05). After applying BAY-11-7082, the content of phosphorylated IκB-α in cell and phosphorylated NF-κB-P65 in its nucleus were significantly reduced (P<0.05), and mRNA and protein levels of NLRP3, pro-caspase-1, pro-IL-18, pro-IL-1β were also significantly reduced (P<0.05). At the same time, the protein content of activated caspase-1 and the secretion of IL-18 and IL-1β in the supernatant were also significantly reduced (P<0.05). Finally, we measured the content of LDH in microglia and their cell survival rate. Compared with the co-culture group, after applying BAY-11-7082, the content of LDH in microglia decreased and the survival rate of cells increased (P<0.05).

(2) In the process of co-cultivation, DEX and RU486 could intervene the NLRP3 inflammasome in microglia by affecting the NF-κB pathway.

As show in **Figure 3**, we then used DEX and glucocorticoid receptor (GR) inhibitor RU486 to intervene in the co-culture process separately. After 4 hours of co-culture, cells and supernatant were collected and observed. The results showed that, DEX could play a similar effect as IκB-α inhibitor BAY117082 (P<0.05). Compared with the Co-culture group, the content of phosphorylated IκB-α in cell and phosphorylated NF-κB-P65 in the nucleus were both significantly reduced (P<0.05), and the mRNA and protein levels of NLRP3 and pro-caspase-1, pro-IL-18, pro-IL-1β were also significantly reduced (P<0.05). At the same time, the protein content of activated caspase-1 in microglia and the secretion of IL-18 and IL-1β in the supernatant were also significantly reduced (P<0.05). Finally, we measured the content of LDH in microglia and their cell survival rate. It suggested that compared with the co-culture group, after applying DEX, the content of LDH in microglia decreased and the survival rate of cells increased (P<0.05). Subsequently, we intervened the co-culture process with the RU486 and DEX at the same time, and observed the pathways of NF-κB and NLRP3 inflammasome. The results showed that after combined application, the content of phosphorylated IκB-α in cell and phosphorylated NF-κB-P65 in the nucleus, and the activation degree of NLRP3 inflammasome pathway were not significantly different from the co-culture group (P>0.05).

### During the Co-Cultivation, the Activation of NLRP3 Inflammasome in Microglia Involved P2X7R Pathway

(1) After co-cultivation, the expression of P2X7R in microglia increased, and P2X7R inhibitor BBG had an inhibitory effect on NLRP3 inflammasome pathway.

**Figure 4:**
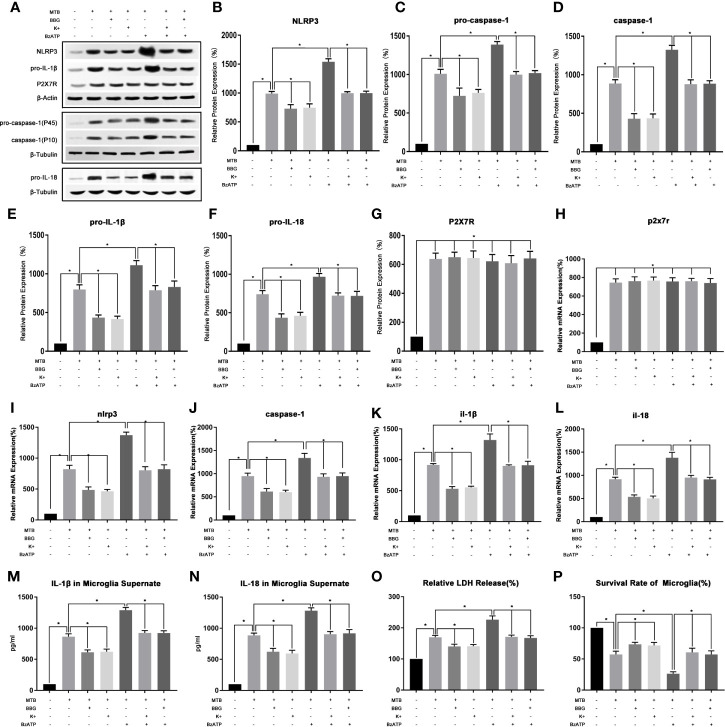
In the process of co-culture, the changes of P2X7R pathway and its intervention factors during the activition of NLRP3 inflammasome in microglia. **(A–G)** Use Western Blot to detect the relative protein expression of NLRP3, pro-caspase-1, caspase-1, pro-IL-1β and pro-IL-18, and P2X7R in control group, co-culture group, BBG group, K^+^ group, BzATP group, BBG+BzATP group and K^+^+BzATP group. **(H–L)** Use Real-time PCR to detect the relative mRNA expression of nlrp3, caspase-1, il-1β and il-18, and p2x7r in control group, co-culture group, BBG group, K^+^ group, BzATP group, BBG+BzATP group and K^+^+BzATP group. **(M, N)** Use ELISA to detect the IL-1β and IL-18 secretion in supernatant of control group, co-culture group, BBG group, K^+^ group, BzATP group, BBG+BzATP group and K^+^+BzATP group. **(O)** Use LDH release to measure the cytotoxicity in control group, co-culture group, BBG group, K^+^ group, BzATP group, BBG+BzATP group and K^+^+BzATP group. **(P)** Use MTT analysis to detect the cell survival rate in control group, co-culture group, BBG group, K^+^ group, BzATP group, BBG+BzATP group and K^+^+BzATP group. *P < 0.05.

As show in **Figure 4**, we first examined the expression level of P2X7R in microglia after co-culture. The results showed that compared with the control group, the mRNA and protein content of P2X7R in co-culture group were significantly increased (P<0.05). After applying BBG, compared with the co-culture group, the mRNA and protein content of P2X7R did not change significantly (P>0.05), but the mRNA and protein content of NLRP3 and pro-caspase-1, pro-IL-18 and pro-IL-1β were significantly reduced (P<0.05). At the same time, the protein content of activated caspase-1 and the secretion of IL-18 and IL-1β were also significantly reduced (P<0.05). Finally, we measured the content of LDH in microglia and their cell survival rate, compared with the co-culture group, after applying BBG, the content of LDH in microglia decreased and the survival rate of cells increased (P<0.05).

(2) Intervention with P2X7R could regulate NLRP3 inflammasome in microglia during co-culture process.

As show in **Figure 4**, we created an extracellular potassium environment (K+) in the co-culture medium to observe the changes in P2X7R and NLRP3 inflammatory. The results showed that the extracellular potassium environment could play a similar effect to the P2X7R inhibitor BBG (P>0.05). Compared with the co-culture group, there was no significant change in the mRNA and protein content of P2X7R in K+ group(P>0.05). However, the mRNA and protein content of NLRP3 and pro-caspase-1, pro-IL-18, and pro-IL-1β were significantly reduced (P<0.05). At the same time, the protein content of activated caspase-1 and the secretion of IL-18 and IL-1β were also significantly reduced (P<0.05). Finally, we measured the content of LDH in microglia and their cell survival rate, compared with the co-culture group, the content of LDH in microglia decreased and the survival rate of cells increased in the K+ group (P<0.05).

Then we applied the P2X7R agonist BzAPT to intervene in the co-culture process, and observe the P2X7R and NLRP3 inflammasome. The results showed that compared with the co-culture group, there was no significant change in the mRNA and protein content of P2X7R after applying BzAPT (P>0.0.5). But the mRNA and protein content of NLRP3, pro-caspase-1, pro-IL-18 and pro-IL-1β increased significantly (P <0.05). At the same time, the protein content of activated caspase-1, and the secretion of IL-18 and IL-1β were also increased significantly (P<0.05). Finally, we measured the content of LDH in microglia and their cell survival rate, compared with the co-culture group, after applying BzAPT, the content of LDH in microglia increased significantly, while the cell survival rate decreased (P<0.05).

Finally, BBG combined with BzATP, as well as extracellular high potassium combined with BzAPT were used to intervene in the co-culture process, and the changes in P2X7R and NLRP3 inflammasome was observed. The experimental results showed that after combined application, the activation of P2X7R and NLRP3 inflammasome was similar to the co-culture group (P>0.05).

## Discussion

In this study, we used a co-culture model of microglia and H37Ra to simulate tuberculosis infection in the CNS, and studied whether there is activation of the NLRP3 inflammasome pathway in microglia during this process, and what are its intervention factors. The results indicated that during the co-cultivation process, the NLRP3 inflammasome and its downstream molecules in microglia activated, the gene transcription and protein expression of the involved molecules increased significantly. Finally, the secreted IL-18 and IL-1β, the LDH, and the mortality of microglia were also increased. By interfering with the functions of NLRP3 and caspase-1, or by interfering with NF-κB in signal 1, or by interfering with P2X7R in signal 2, the secretion of IL-18, IL-1β and the death of microglia could be reduced.

*Mtb* infection of the CNS involves a series of complex pathophysiological processes, among which excessive inflammation is an important cause of brain damage. Microglia are vital immune cells in the CNS and involved in many aspects of the inflammatory response, they play an important role in the process of tuberculosis infection in the CNS (Rock et al., 2004; Spanos et al., 2015). A number of previous studies have shown that there are many different inflammasomes in microglia. These inflammasomes are widely involved in many different types of neurological diseases, especially in neurological infectious diseases (Marim et al., 2017; Heneka et al., 2018; Katuri et al., 2019; Xue et al., 2019; Cui et al., 2020; Ji et al., 2020; Ma et al., 2020; Zeng et al., 2020). Lee et al. (2013) have found that after co-cultivation with *Mtb* and macrophages, the culture supernatant can activate the NLRP3 inflammasome pathway in microglia. Our experiment had similarities with it. The difference was that we used *Mtb* to directly interact with microglia to observe its effect on NLRP3 inflammasomes in microglia. This is because microglia and macrophages are homologous. They enter the CNS during development and eventually become macrophages that reside in the CNS. Their immune response and inflammatory response are closely related to the CNS, which is also the same in the process of CNS *Mtb* infection. Therefore, we made *Mtb* interact directly with microglia, and observed the influence of *Mtb* on the inflammatory response of microglia in a more intuitive way.

Our experiments suggested that after *Mtb* interacts with microglia, microglia produced and released a large number of inflammatory factors through the NLRP3 inflammasome pathway, which promoted cell death. By regulating this process could reduce the inflammatory response of microglia to a certain extent. Our experiment is of great significance to clarify the mechanism of inflammatory response and to regulate excessive inflammatory response in CNS tuberculosis infection.

As mentioned above, the classic activation of NLRP3 inflammasome is divided into two stages: signal 1 and signal 2. We first observed the NF-κB pathway in signal1. Previous research has shown that with the phosphorylation of IκB-α, IκB dissociates from NF-κB and moves to the proteasome and degrades, thereby translocating NF-κB to the nucleus (Kharytonchyk et al., 2018). By using BAY-11-7082 to regulate the phosphorylation of IκB-α located upstream of NF-κB, it could affect the nuclear translocation of NF-κB and reduce the phosphorylated NFκB-P65 in the nucleus, thereby reduced the gene transcription of each molecule in the NLRP3 inflammasome pathway, and ultimately reduced the secretion of IL-18 and IL-1β. This confirms that in the process of tuberculosis infection in the CNS, the activation of NLRP3 inflammasomes in microglial is mediated by NF-κB, and the regulation of NLRP3 inflammasomes can be achieved by intervening in the function of NF-κB. Subsequently, we intervened in the co-culture process with DEX, a drug that is clinically used to alleviate the inflammatory response of tuberculosis infection in the CNS (Shane and Riley, 1953; Thwaites et al., 2004), to understand whether it has a regulatory effect on NRLP3 inflammasome. Gräb et al. (Gräb et al., 2019) has found that corticosteroids such as DEX inhibit necrotic cell death of cells infected with *Mtb* by facilitating mitogen-activated protein kinase phosphatase 1 (MKP-1)-dependent dephosphorylation of p38 MAPK. Green et.al (Green et al., 2010) has found that *Mtb* can upregulates microglial matrix metalloproteinase-1 and -3 expression and secretion *via* NF-κB and activator Protein-1-dependent monocyte networks. And in this study, we found that DEX can affect the phosphorylation of IκB-α through GR receptors, thereby exerting a similar effect to BAY-11-7082, affecting the transcription of various molecules in the NLRP3 inflammasome pathway, and ultimately reducing the release of IL-18 and IL-1β, alleviate cytotoxicity and cell death. After applying the GR receptor inhibitor RU486, the above-mentioned effects of DEX almost disappeared. These results are consisted with previous studies (Oh et al., 2017; Seshadri et al., 2018) that GR can bind to IκB and inhibit its phosphorylation after being activated, thereby inhibiting the nuclear translocation of NF-κB and its function in the nucleus. In addition, there are also studies showed that DEX can inhibit the activation of NLRP3 inflammasomes in other different types of cells (Cao et al., 2018; Pellegrini et al., 2018; Gong et al., 2019; Yang et al., 2019). So, our experiment supports the rationality of the application of glucocorticoids in the clinical treatment of tuberculosis infection of the CNS.

In addition, the process of CNS *Mtb* infection is often accompanied by cell energy metabolism disorders, and the imbalance of sodium and potassium ions inside and outside the cell (Fernando et al., 2007; Davis et al., 2019). In addition, as nerve cells are continuously invaded by *Mtb*, the integrity of the cell membrane is destroyed, and a large number of intracellular substances including potassium ions and ATP are released (Fernando et al., 2007; Davis et al., 2019). Therefore, we chose P2X7R in signal2 for observation. P2X7R is a non-specific cation channel that can be activated by ATP released by cell death, and its function is also highly dependent on the outflow of potassium ions, its function is closely related to the assembly of NLRP3 inflammasome complex (Karmakar et al., 2016). P2X7R in microglia is widely involved in the process of many neurological diseases (Franceschini et al., 2015; Yue et al., 2017; Zhao et al., 2017), We first observed the changes of P2X7R during the co-cultivation process and found that there was indeed a significant up-regulation of P2X7R expression. Subsequently, we used BBG to inhibit the function of P2X7R and found that it could significantly reduce the expression of NLRP3 inflammasome-related molecules, and reduce the final release of IL-18 and IL-1β, suggesting that P2X7R plays an important role in the activation of NLRP3 in microglia. Subsequently, we created an extracellular high potassium environment to inhibit the outflow of potassium ions, and found that it can play a similar role to the P2X7R inhibitor BBG, and the application of BzATP could significantly enhance the effect of P2X7R and enhance the NLRP3 inflammasome pathway, and increase the release of inflammatory factors. The above experimental results indicate that in the process of tuberculosis infection in the CNS, the imbalance of intracellular and extracellular electrolytes, as well as the APT released by necrotic cells, can affect the P2X7R receptor on the microglia membrane and further lead to the NLRP3 inflammasome pathway activation and the release of inflammatory factors. These inflammatory factors can cause a subsequent cascade reaction and cause more serious nerve cell damage. This also explains from one perspective why such a severe inflammatory response occurs after the CNS is infected with *Mtb*.

However, this experiment also has some shortcomings. First of all, we did not clarify which bacterial components of *Mtb* interacted with microglia. For example, Liu et al. (2021) found that lipoprotein LpqH in *Mtb* can active NLRP3 inflammasome in mouse Ana-1 macrophage. Also, Beckwith et al. (2020) has found that plasma membrane damage causes NLRP3 activation and pyroptosis during *Mtb* infection. But as composition of *Mtb* is very complex, and a complex immune cascade also occurs during the infection process, we have not been able to determine the specific components that can activate microglia. In addition, there are a variety of inflammasomes in microglia, which are involved in many different kinds of neurological diseases (Marim et al., 2017; Heneka et al., 2018). However, in this study, we did not conduct further research on this, and it is not ruled out that other types of inflammasomes participate in the co-culture process. Finally, in this study, we did not study the relationship between P2X7R and NFκB, and the reasons why P2X7R caused gene transcription in changes in NLRP3 inflammasome-related moleculars. Previous studies have shown that P2X7R not only affects the assembly of NLRP3 inflammasomes, but also affects the transcription of related molecules (Ferrari et al., 2006; Potucek et al., 2006; Mingam et al., 2008; Piccini et al., 2008). Also, there is a close relationship between P2X7R and NF-κB (Yu et al., 2019; Yang et al., 2020). Therefore, the regulation of P2X7R may further affect the gene transcription process through the NF-κB pathway. This may also be one of the deeper mechanisms that P2X7R regulates NLRP3 inflammasome-related molecules. The above-mentioned problems will be further confirmed in follow-up research.

## Conclusion

Through this study, we confirm for the first time that NLRP3 inflammasome and its downstream pathways are activated in the *Mtb* H37Ra strain and rat microglia co-culture model. The activation of NLRP3 inflammasome is closely related to the NF-κB pathway in signal1 and the P2X7R in signal2. Interventions on these can regulate the function of the NLRP3 inflammasome pathway and affect the production of inflammatory factors, cytotoxicity and cell survival in microglia. Using H37Ra and microglia co-culture model to study the *Mtb* infection in CNS can further understand its pathogenesis and provide a useful reference for its treatment.

## Data Availability Statement

The raw data supporting the conclusions of this article will be made available by the authors, without undue reservation.

## Ethics Statement

The animal study was reviewed and approved by Animal Care and Use Committee of the Northwest University.

## Author Contributions

ZX is responsible for experimental design and proteomics experiments. HH is responsible for genomics experiments. QY and YD are responsible for cultivation of cells and bacteria. WL, YC, and MZ are in charge of ELIAS experiments and the collection and sorting of documents. GZ and YT are responsible for data sorting and analysis. ZX was a major contributor in writing the manuscript. All authors contributed to the article and approved the submitted version.

## Funding

This work was supported by grants from: 1) Natural Science Basic Research Program of Shaanxi (Program No. 2019JQ-251) and Natural Science Basic Research Program of Shaanxi (Program No. 2019JQ-543), supported the design of the study and the collection of date. 2) National Natural Science Foundation of China (Program No. 81671185), supported in the reagent purchase and instrument usage cost. 3) Hospital-level project of Xi’an International Medical Center (Program No. 2020ZD007) and Natural Science Basic Research Program of Xi’an (Program No. 2020qn17), supported in the expenses related to laboratory animals and cell culture. 4) the Key Research and Development Program of Shaanxi (Program No.2020ZDLSF04-03), supported in the analysis, and interpretation of data. 5) Science and Technology Project of Xi’an, Shaanxi Province, China 201805104YX12SF38(2), supported in writing the manuscript.

## Conflict of Interest

The authors declare that the research was conducted in the absence of any commercial or financial relationships that could be construed as a potential conflict of interest.

## Abbreviations

CNS, centrol neuron system; TM, Tuberculous meningitis; *Mtb*, Mycobacterium tuberculosis; P2X7R, P2X7 receptor; DMSO, Dimethylsulfoxide; K^+^, potassium ion solution; GR, Glucocorticoid receptor; DEX, Dexamethasone; LDH, lactate dehydrogenase enzyme.
